# Gorlin-Goltz Syndrome: A Rare Case Report of a 11-Year-Old Child

**DOI:** 10.5005/jp-journals-10005-1374

**Published:** 2016-09-27

**Authors:** Sandeep Tandon, Yashwant Chauhan, Meenakshi Sharma, Manish Jain

**Affiliations:** 1 Senior Professor and Head, Department of Pedodontics and Preventive Dentistry, RUHS College of Dental Sciences, Jaipur, Rajasthan, India; 2 Postgraduate Student, Department of Pedodontics and Preventive Dentistry, RUHS College of Dental Sciences, Jaipur, Rajasthan, India; 3 Senior Lecturer, Department of Pedodontics and Preventive Dentistry, RUHS College of Dental Sciences, Jaipur, Rajasthan, India; 4 Associate Professor, Department of Oral Pathology, NIMS Dental College, Jaipur Rajasthan, India

**Keywords:** Gorlin-Goltz syndrome, Nevoid basal cell carcinoma, Odontogenic keratocysts.

## Abstract

Gorlin-Goltz Syndrome, also known as nevoid basal cell carcinoma syndrome (NBCCS), is an autosomal dominant trait caused due to mutations in the patched tumor suppressor gene (PTCH) gene found on the long arm of chromosome 9. The syndrome is characterized by the presence of odontogenic keratocysts (OKCs), basal cell carcinomas, and skeletal malformations. Early diagnosis of the syndrome can be done by pedodontist as OKC is one of the early manifestations of the syndrome. Early diagnosis and treatment is important for long-term prognosis of the syndrome by reducing the severity of cutaneous carcinomas and deformities due to jaw cyst. The present case describes an 11-year-old patient with some typical features of NBCCS, which were diagnosed through its oral and maxillofacial manifestations. This case emphasizes the importance of pedodontist in early recognition of the syndrome.

**How to cite this article:**

Tandon S, Chauhan Y, Sharma M, Jain M. Gorlin-Goltz Syndrome: A Rare Case Report of a 11-Year-Old Child. Int J Clin Pediatr Dent 2016;9(3):264-268.

## INTRODUCTION

Gorlin-Goltz syndrome or nevoid basal cell carcinoma syndrome (NBCCS) was described by Gorlin and Goltz in 1960.^1^ It is an autosomal dominant disorder with a high degree of penetrance and variable expressivity.^2^ The incidence of this disorder is estimated to be 1 in 50,000 to 150,000 in general population, varying by region.^3^ However, individual features of this disorder have been described for centuries with the first evidence in historical document from ancient Egypt. The syndrome was first described by Jarish and White in 1894 as they noticed the presence of multiple basocellular carcinoma, and then in 1960 Gorlin and Goltz described classical triad of multiple basocellular epithilioma, keratocyst in jaw, and bifid rib, which was later on modified by Rayner et al^4^ in 1977, who established that for diagnosis at least cyst had to appear in combination with calcification of the falx cerebri or palmer and planter pits.

Pathogenesis of NBCCS is due to mutations in the patched tumor suppressor gene (PTCH) on chromosome 9q21-23 where abnormality in the Hedgehog (Hh) signaling pathway results in neoplasm formation.^5^

Early diagnosis of the syndrome is of great clinical importance since severity of complications, such as maxilloacial deformities related to the jaw cyst can be avoided and long-term prognosis of malignant skin lesion and brain tumor is better when early diagnosis and treatment can be initiated.^6^

The present case describes a patient with some typical features of NBCCS, which were diagnosed for the first time in our department. Furthermore, the case emphasizes the importance of pedodontist in early recognition of the syndrome.

## CASE REPORT

An 11-year-old young patient reported to Department of Pedodontics and Preventive Dentistry, Government Dental College and Hospital, Jaipur, Rajasthan, India, with chief complaint of slowly growing swelling on right lower posterior side of face for 3 months. Swelling was associated with dull pain which was localized, and not with ulceration, inflammation, or paresthesia.

Patient had natal history of birth hypoxia with hypoxic ischemic encephalopathy (HIE) which was resolved after 1 year according to computed tomography (CT) report, and the patient’s mother was operated for odontogenic keratocyst (OKC).

**Fig. 1:**
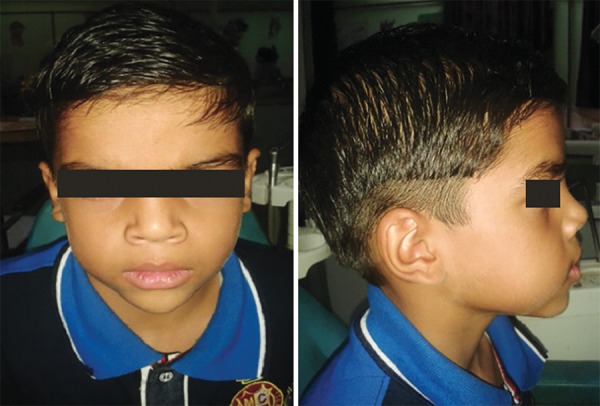
Preoperative frontal and lateral profile of patient

On extraoral clinical examination, a hard, tender swelling of about 3.5 × 3 cm with ill-defined borders on right mandibular premolar region was present. There was no lymphadenopathy (Fig. 1).

Intraorally, the swelling was associated with expansion of buccal cortical plate with obliteration of vestibule adjacent to right lower first molar and egg shell crackling. There was no mobility or pain on percussion of associated teeth. The left-side extra- and intraoral examination did not revealed any significant details.

**Fig. 2:**
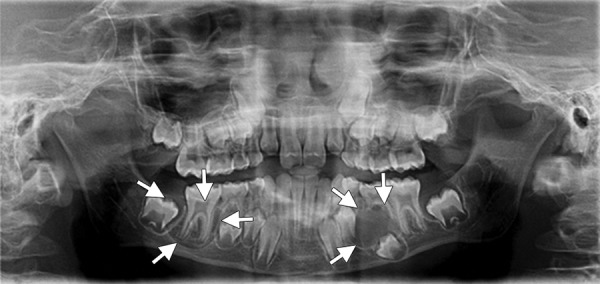
Preoperative OPG showing bilateral OKCs in posterior mandible

On radiographic examination, orthopantomography (OPG) showed bilateral, well-defined radiolucencies surrounded by corticated, scalloped radiopaque borders giving multilocular appearance (Fig. 2).

**Fig. 3:**
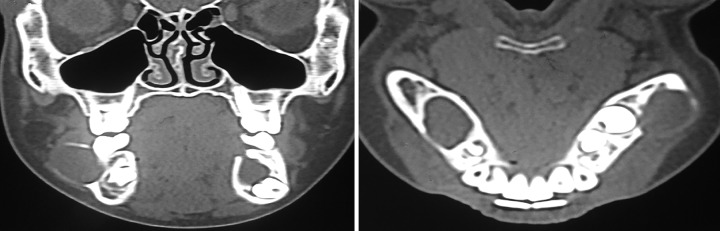
Computed tomography mandible showing bilateral OKCs

**Fig. 4:**
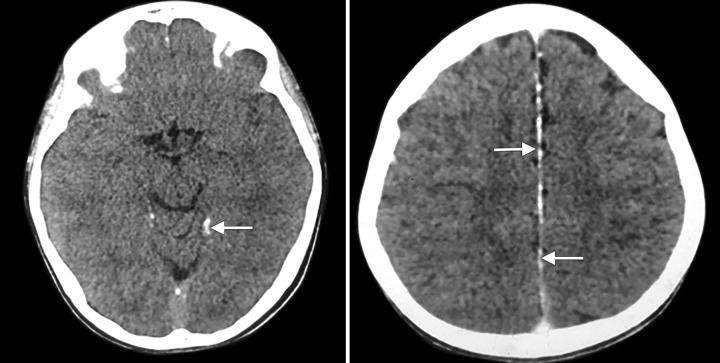
Computed tomography brain showing calcification of falx cerebri and tentorium

Computed tomography images revealed abnormal multilocular, expansile, cystic lesion with a bony sclerotic margin (Fig. 3). Owing to the presence of bilateral cystic swelling in mandible, Gorlin-Goltz syndrome was suspected and further investigations were carried out.

**Fig. 5:**
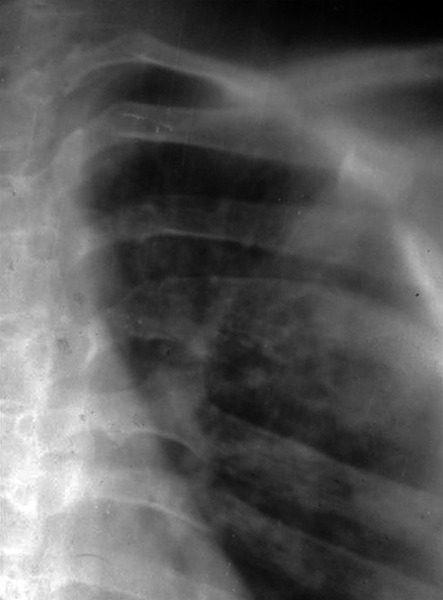
Chest radiograph showing unilateral bifid 5th rib

Computed tomography/radiograph report of our case revealed:

- Bilateral OKCs in mandibular molar region
- Calcification of falx cerebri
- Chest radiograph bifid rib was present (Figs 4 and 5)

**Fig. 6:**
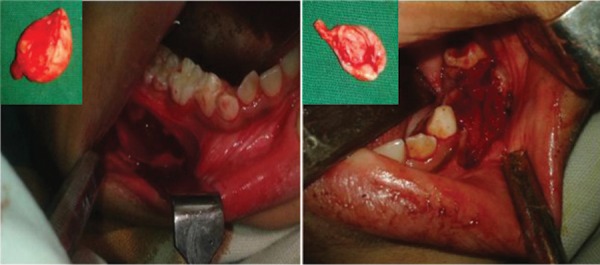
Intraoperative view with enucleated cysts

**Fig. 7:**
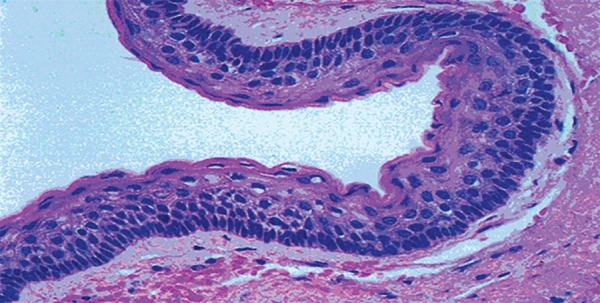
Micrograph of OKC showing hyperchromatism and pallisading appearance

**Fig. 8:**
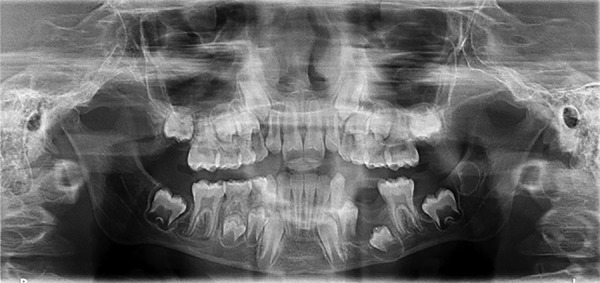
Orthopantomography at 3-month follow-up

**Fig. 9:**
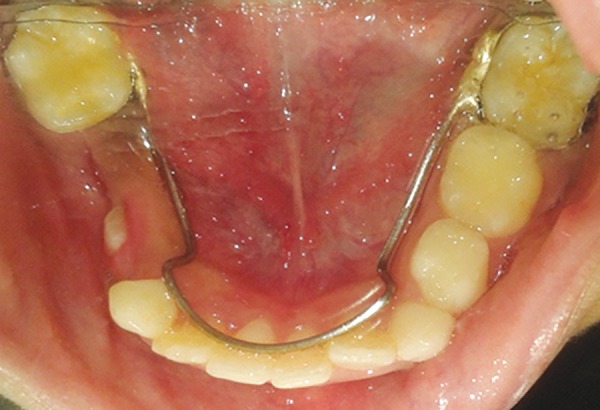
Mandibular occlusal view with lingual arch space maintainer

The case is diagnosed using diagnostic protocol of Lo Muzio.^7^ Treatment plan included enucleation under general anesthesia.

- Procedure on right side - enucleation followed by primary closure.
- Procedure on left side - enucleation with fenestration or open packing with iodoform gauge, after extraction of 74 and 75 (Fig. 6).

Histopathologically the report showed parakera-tinized stratified epithelium with an average thickness of 5 to 8 cells, with basal cells fenced up in a corrugated surface and a connective wall rich in mucopolisa-carides and with a variable number of microcysts and epithelial islets seen. This confirmed the OKCs on both sides (Fig. 7).

The patient was on regular follow-up (Fig. 8). Lingual arch space maintainer was given for smooth eruption of 34 and 35 (Fig. 9).

## DISCUSSION

Diagnostic criteria for NBCCS was established by Evans et al,^8^ modified by Kimonis et al,^9^ reviewed by Manfredi et al,^10^ and more recently, a multi disciplinary colloquium was organized to better define the physical findings associated with NBCCS. The participants reviewed the diagnostic criteria of the syndrome, and there was no consensus for a formal recommendation. Consequently, a suspected diagnosis of NBCCS should be considered based on findings of less stringent criteria:

- One major criterion and molecular confirmation
- Two major criteria
- One major and two minor criteria

**Table 1:** **Table 1:** Major and minor criteria stated by first international colloquium on NBCCS

*Sl. no.*		*Major criteria*		*Minor criteria*	
1		Basal cell carcinoma (BCC) prior to 20 years old or excessive number		Rib anomalies	
		of BCC out of proportion to prior sun exposure and skin type			
2		Odontogenic keratocyst of jaw prior to 20 years of age		Macrocephaly determined after adjustment for height	
3		Palmer or planter pitting		Other specific skeletal malformation and radiologic	
				changes (i.e., vertebral anomalies, kyphoscoliosis,	
				short fourth metacarpals, past axial polyductyly)	
4		Lamellar calcification of the falx cerebri		Cleft lip/palate	
5		Medulloblastoma, typically desmoplastic		Ovarian/cardiac fibroma	
6		First degree in relation to NBCCS		Lymphomesentric cysts	
				Ocular abnormalities (i.e., strasbismus, hypertelorism,	
				congenital cataract, glaucoma, coloboma)	

Major and minor criteria stated by first international colloquium on NBCCS (see Bree et al^11^) is given in Table 1.

In this case, we found two major criteria, i.e., histologically proven OKCs bilaterally in molar region of mandible, calcification of falx cerebri in CT report, and one minor criterion, i.e., unilateral 5th bifid rib. So, this case fits into criteria of Gorlin-Goltz syndrome.

Odontogenic keratocysts are most frequently observed and usually the first manifestation of NBCCS, so they often occur in the early decades of life^12,13^ and have high recurrence rate and tendency toward multiplicity, particularly when associated with NBCCS.^13^ Odontogenic keratocysts, found in posterior molar region of mandible, were supported by studies done by Woolgar et al^14^ The posterior area of mandible was the main affected site, followed by maxillary molar region. Moreover, OKCs were bilateral (i.e., two in number) which were supported by studies by Ahn et al,^15^ who found 1 to 6 OKCs associated with NBCCS cases, and by Gupta et al^16^ who found in a series of case reports six Indian patients, all developed multiple OKCs (ranges from 3-6).

Treatment of OKCs by enucleation may be considered adequate provided that all teeth included within or in contact with the lesion are extracted. However, this treatment can be combined with fenestration or open packing as required depending on size of the lesion and patient’s age. In particular, if the patient is in the first or second decade of life with hitherto unerupted permanent teeth involving OKCs, it would be difficult to make a decision regarding aggressive surgery. Some authors describe how aggressive surgery can have adverse effect on dental development of the affected jaw.^17^ Therefore, in this case, conservative treatment was chosen as on right-side enucleation with closure by suture and on leftside enucleation with fenestration or open packing with iodoform gauge. Also, patients suffering from NBCCS have to undergo checkups at least once a year, especially those having OKCs.^18^

In NBCCS in the skull, there is early onset of calcification with lamellar calcification of falx cerebri up to 70 to 85%, calcification of tentorium cerebelli up to 20%, and dura and choroids.^19^ In this case also, calcification of falx cerebri and tentorium was observed in CT report.

Rib anomaly found in this case was consistent with findings from Shanley et al^20^ and Kimonis et al.^9^

As the name of syndrome is NBCCS, no BCC was found. This can be explained with the help of the studies done by Endo et al^21^ and Ahn et al^15^ that BCC prevalence seems to be low in Asia, and BCC proliferates between puberty and 35 years of age.^22^ Because, in this case, the patient is 11 years old, we suggest continuous monitoring as there may be possibility of BCC occurrence in future.

The guidelines for follow-up of NBCCS as given by de Amezaga et al^6^ should be followed:

- Neurological examination - twice yearly
- Cerebral MRI - once in year for 1 to 7 years of age
- Skin examination - yearly
- Cardiologic examination - according to sign and symptoms
- Genetic counseling of families as it is an autosomal dominant disorder.

## CONCLUSION

This case shows the importance of awareness of this rare syndrome in young people without any skin lesions. Early diagnosis of syndrome and a long follow-up period is important due to the severity of clinical manifestation. Moreover, a multidisciplinary team is required, including dentist, dermatologist, geneticists, and neurologist, so that there are increased chances of better overall survival rates. So Gorlin-Goltz syndrome was diagnosed and treated for one of its major anomalies, i.e., OKCs, whereas the other anomalies did not require active management at this stage. Lingual arch space maintainer was given for smooth eruption of 34 and 35.
